# Oxidation of the FAD cofactor to the 8-formyl-derivative in human electron-transferring flavoprotein

**DOI:** 10.1074/jbc.RA117.000846

**Published:** 2018-01-04

**Authors:** Peter Augustin, Marina Toplak, Katharina Fuchs, Eva Christine Gerstmann, Ruth Prassl, Andreas Winkler, Peter Macheroux

**Affiliations:** From the ‡ Institute of Biochemistry, Graz University of Technology, Petersgasse 12/II and; the § Institute of Biophysics, Medical University of Graz, Neue Stiftingtalstrasse 6/IV, 8010 Graz, Austria

**Keywords:** dehydrogenase, mitochondria, electron transfer, flavin adenine dinucleotide (FAD), respiratory chain, 8-formyl-FAD, flavin semiquinone

## Abstract

The heterodimeric human (h) electron-transferring flavoprotein (ETF) transfers electrons from at least 13 different flavin dehydrogenases to the mitochondrial respiratory chain through a non-covalently bound FAD cofactor. Here, we describe the discovery of an irreversible and pH-dependent oxidation of the 8α-methyl group to 8-formyl-FAD (8f-FAD), which represents a unique chemical modification of a flavin cofactor in the human flavoproteome. Furthermore, a set of hETF variants revealed that several conserved amino acid residues in the FAD-binding pocket of electron-transferring flavoproteins are required for the conversion to the formyl group. Two of the variants generated in our study, namely αR249C and αT266M, cause glutaric aciduria type II, a severe inherited disease. Both of the variants showed impaired formation of 8f-FAD shedding new light on the potential molecular cause of disease development. Interestingly, the conversion of FAD to 8f-FAD yields a very stable flavin semiquinone that exhibited slightly lower rates of electron transfer in an artificial assay system than hETF containing FAD. In contrast, the formation of 8f-FAD enhanced the affinity to human dimethylglycine dehydrogenase 5-fold, indicating that formation of 8f-FAD modulates the interaction of hETF with client enzymes in the mitochondrial matrix. Thus, we hypothesize that the FAD cofactor bound to hETF is subject to oxidation in the alkaline (pH 8) environment of the mitochondrial matrix, which may modulate electron transport between client dehydrogenases and the respiratory chain. This discovery challenges the current concepts of electron transfer processes in mitochondria.

## Introduction

**Scheme 1.**
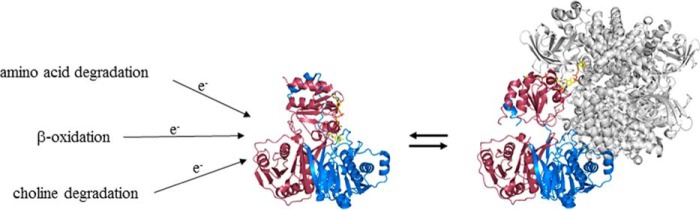
**Interaction of human flavin dehydrogenases with hETF.** So far, 13 flavin dehydrogenases, involved in β-oxidation (short chain acyl-CoA dehydrogenase; medium chain acyl-CoA dehydrogenase; long chain acyl-CoA dehydrogenase; very long chain acyl-CoA dehydrogenase; and acyl-CoA dehydrogenase family member 9–11), amino acid (short branched chain acyl-CoA, iso-valeryl-CoA, iso-butyryl-CoA, and glutaryl-CoA dehydrogenase), and choline degradation (dimethylglycine and sarcosine dehydrogenase) were identified to interact with hETF. hETF exhibits a flexible interaction mechanism and adopts a closed non-productive form (PDB code 1EFV) and an open productive conformation, here shown bound to human medium chain acyl-CoA dehydrogenase (PDB code 2A1T). The α- and β-subunits of hETF are shown in *raspberry* and in *marine* cartoon view, respectively. hETF bound hMCAD is displayed in *gray*. hETF bound FAD is presented in *yellow sticks*, hMCAD bound FAD in *pink stick* representation.

In 1956, Crane *et al.* (1) identified the electron-transferring ability of an unknown flavoprotein from the pig liver, which they named electron-transferring flavoprotein (ETF).^3^ Since then, numerous studies on ETF have been reported, and orthologs have been described in all kingdoms of life (2). The heterodimeric human (h) ETF serves as a central electron carrier in the mitochondrial matrix. hETF accepts electrons from 13 flavin dehydrogenases and transfers them to the human ETF-ubiquinone oxidoreductase (hETF-QO), an iron–sulfur cluster containing flavoprotein bound to the inner mitochondrial membrane that feeds these electrons into the mitochondrial respiratory chain (2, 3). The flavin dehydrogenases are either part of β-oxidation, amino acid, or choline degradation, as shown in Scheme 1.

Interestingly, these dehydrogenases are structurally distinct with dehydrogenases operating either in the degradation of fatty or amino acids adopting the “acyl-CoA dehydrogenase”-fold, whereas both dimethylglycine dehydrogenase (hDMGDH) and sarcosine dehydrogenase are part of the amine oxidase protein family. Apparently, hETF has evolved a flexible mechanism to interact with various dehydrogenases as well as with hETF-QO (2). The protein exists in a closed non-productive and in an open productive conformation with a highly flexible upper protein domain (Scheme 1). The interaction with client dehydrogenases is initiated by a recognition peptide of the β-subunit leading to the exposure of the FAD and concomitant electron transfer (4).

Recently, Toogood *et al.* (4) have shown that a single amino acid replacement in the β-subunit (βE165A) favors the open conformation by removing a side-chain interaction with αAsn-259 in the α-subunit. As a consequence, variant βE165A displayed higher affinity to the human medium-chain acyl-CoA dehydrogenase (hMCAD) (and to rat DMGDH) enabling co-crystallization (Scheme 1). In the course of our attempts to co-crystallize hETF with hDMGDH, we have reproduced the βE165A variant as well as generated the corresponding αN259A variant. However, characterization of the variants by UV-visible absorption spectroscopy indicated that the FAD cofactor undergoes a slow conversion affecting the isoalloxazine ring. A detailed analysis of the isolated flavin moiety showed that the observed conversion entails the oxidation of the 8α-methyl to the formyl group, *i.e.* 8-formyl-FAD (8f-FAD) is the product of the spontaneous conversion observed in these two variants. This prompted us to extend the study to include wildtype hETF and several variants with amino acid replacements in the binding pocket of the isoalloxazine ring. In this study, we document that (i) conversion of FAD to 8f-FAD also occurs in wildtype hETF at alkaline pH, (ii) the 8f-FAD stabilizes an oxygen-insensitive semiquinone radical, and (iii) several highly conserved amino acid residues in the FAD-binding pocket are essential for the formation of 8f-FAD. In addition, we demonstrate that wildtype hETF bearing 8f-FAD as cofactor exhibits increased affinity to reduced hDMGDH under steady-state conditions suggesting a potential role *in vivo*. Taken together, our results report entirely novel properties of hETF that may have important implications for our understanding of electron handling in the mitochondrial matrix.

## Results

### Time-dependent absorption changes of purified recombinant hETF

**Figure 1.**
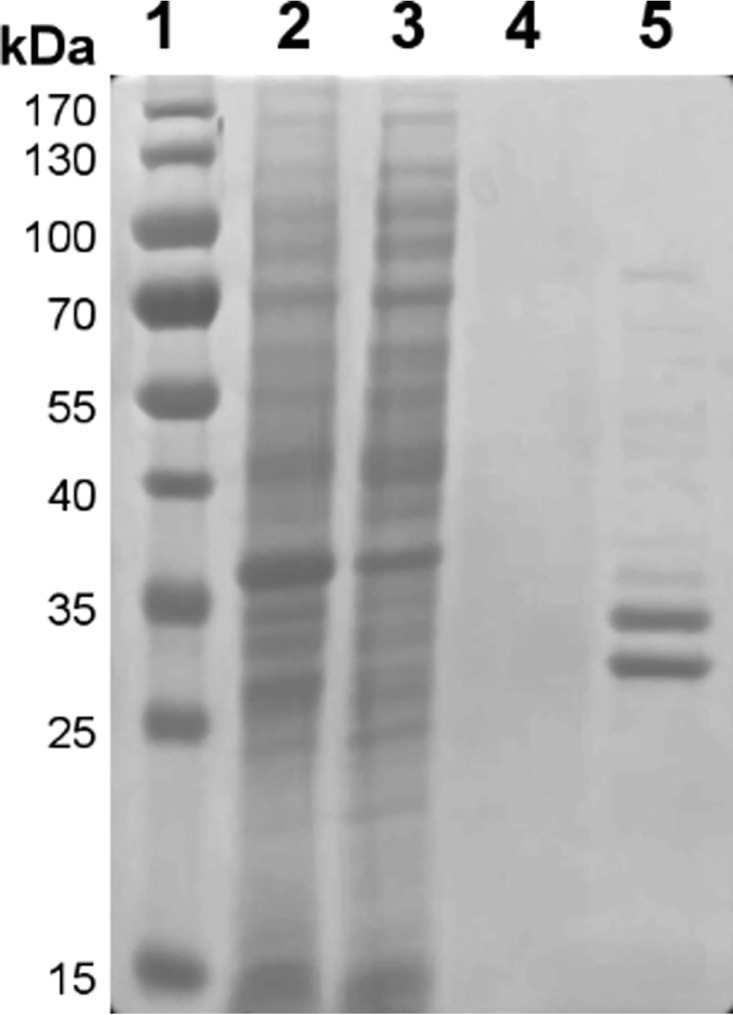
**Purification of hETF-WT.** The SDS-PAGE of hETF Ni-NTA affinity chromatography is shown as follows: *lane 1,* PageRuler® prestained protein ladder (Thermo Fisher Scientific); *lane 2*, cell lysate; *lane 3*, column flow-through; *lane 4*, washing fraction; and *lane 5*, elution fraction.

**Figure 2.**
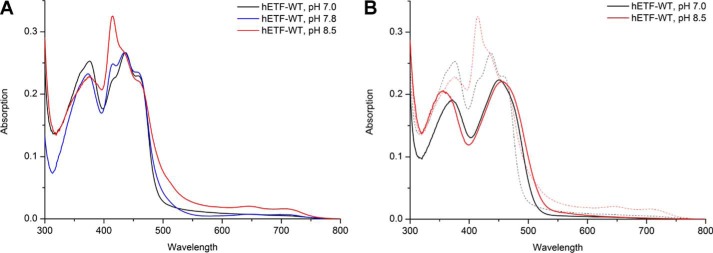
**UV-visible absorption spectra of native (*A*) and denatured hETF-WT (*B*) purified at pH 7 (7. 8) and 8.5.** *A,* pH conditions used in the purification of hETF-WT, pH 7 (*black line*), 7.8 (*blue line*), and 8.5 (*red line*), strongly affected the absorption spectra of the isolated protein. *B,* denaturation of hETF purified at pH 7 (*black*) and 8.5 (*red*) with 20% SDS also resulted in different absorption spectra (*dotted* and *solid lines* represent spectra recorded before and after denaturation, respectively).

hETF produced in *Escherichia coli* host cells was purified by means of Ni-NTA affinity chromatography and yielded 2 mg of homogeneous protein per g of wet cell pellet (Fig. 1). To explore the effect of the pH on protein yield and stability, purifications were performed at pH 7, 7.8, and 8.5. This gave rise to different UV-visible absorption spectra as shown in Fig. 2*A*. At neutral pH, the absorption spectrum featured two maxima at 375 and 436 nm, which were very similar to spectral properties reported for hETF and pig liver ETF, respectively (5, 6). However, pronounced changes were observed when hETF was purified at pH 7.8 or 8.5. These spectra were characterized by a bathochromic shift of the maximum at 436 to 415 nm accompanied by an increase in absorption at a longer wavelength (Fig. 2*A*). To check whether the observed spectral changes were caused by a chemical modification of the FAD cofactor, hETF purified at pH 7 and pH 8.5 was denatured, and absorption spectra were recorded. As shown in Fig. 2*B*, hETF purified at pH 7 yielded the typical absorption spectrum of free FAD with maxima at 370 and 450 nm, whereas the flavin moiety released from hETF purified at pH 8.5 featured spectral shifts to 354 and 456 nm, respectively.

### Isolation and identification of the modified FAD generated at alkaline pH

**Figure 3.**
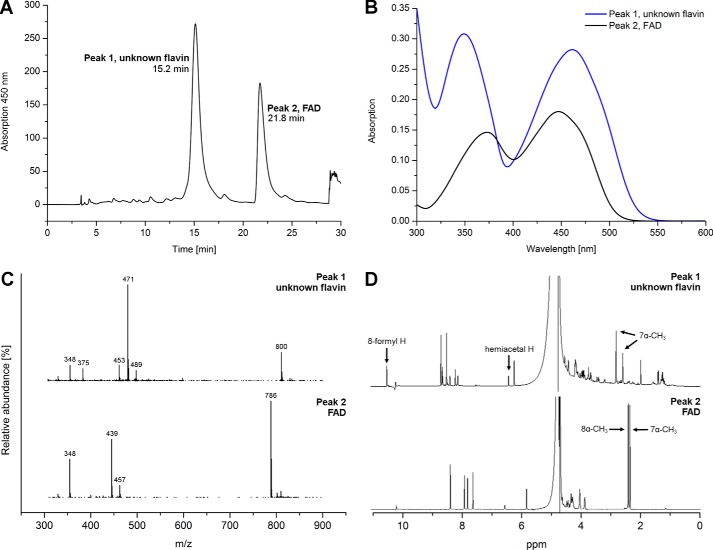
**Analysis and comparison of the two main flavin-containing fractions isolated from hETF.** *A,* HPLC reversed phase purification of the extracted cofactor(s) gave two major fractions, which were further analyzed by MS and NMR. *B,* spectra of the two peaks of HPLC purification featured the same shifts as seen in Fig. 2*B. C,* mass spectra of the two main fractions as separated by HPLC. The spectrum shown at the *bottom* exhibits the typical fragmentation and mass peaks of FAD. The mass spectrum at the *top* shows a mass shift of 14 a.u. *D,* in agreement with the mass analysis, the ^1^H NMR spectrum at the bottom can be assigned to FAD, whereas the additional resonance at 10.4 ppm and the shifts observed for the methyl groups in position 7α and 8α indicate chemical changes in the dimethylbenzene ring moiety of the isoalloxazine ring. Both methods indicate that a small amount of a closed, hemiacetal form of 8f-FAD is present, as observed previously (12).

To identify the nature of the chemical modification in the flavin chromophore, we extracted the cofactor(s) from hETF purified and kept at pH 8.5. After extraction and purification by HPLC (Fig. 3, *A* and *B*), the two main fractions were analyzed by mass spectrometry and NMR spectroscopy (Fig. 3, *C* and *D*, respectively). Mass spectrometric analysis of one of the major fractions (peak 2) clearly showed the typical fragmentation pattern (AMP, *m*/*z* = 348; FMN, *m*/*z* = 439 and 457) and mass of FAD (*m*/*z* = 786; MassBank accession number KNA00248 (7)), whereas the other fraction (peak 1) exhibited a shift of 14 a.u. of all major peaks, except that for AMP. In agreement with the observed differences in the absorption properties, this result confirmed that the chemical modification has most likely occurred in the isoalloxazine ring of the FAD cofactor. Further analysis of the two flavin-containing fractions by NMR spectroscopy revealed the presence of a resonance at ∼10.4 ppm in the unknown flavin species that is absent in FAD. Additional differences were observed in the resonances of the methyl groups at the 7α- and 8α-positions (see *arrows* in Fig. 3*D*). Overall, the ^1^H NMR spectrum possesses the same features as reported previously for 8-formyl-FAD isolated from formate oxidase (8). Taken together with the observed mass difference, we therefore conclude that the 8α-methyl group of FAD was oxidized to a formyl group to yield 8f-FAD.

### Time-dependent formation of 8f-FAD in hETF

**Figure 4.**
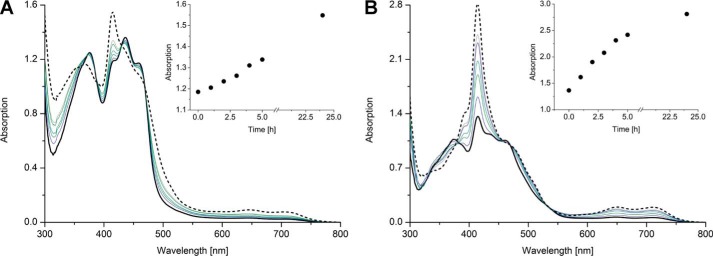
**Formation of 8f-FAD radical in wildtype hETF and the αN259A variant.** *A,* ∼400 μm wildtype hETF purified in 50 mm HEPES, pH 7.0, was diluted 1:20 with HEPES buffer, pH 8.5, and was incubated at 25 °C for 24 h. *B,* ∼400 μm hETF-αN259A purified in 50 mm HEPES, pH 7.0, was diluted 1:20 with HEPES buffer, pH 8.5, and was incubated at 25 °C for 24 h. The *dotted line* in both panels represents the spectrum measured after 24 h. The spectra were normalized to an absorption of 1 at the isosbestic point at 469 nm to simplify comparison. The *insets* show the time-dependent absorption changes recorded at the indicated times at 415 nm.

To obtain further insights into the formation of 8f-FAD in hETF, a sample purified at pH 7 was diluted into buffer at pH 8.5, and the spectral changes were observed over time. As shown in Fig. 4*A*, the formation of 8f-FAD is a slow process with an approximate half-time of 20 h for hETF-WT and 4 h for hETF-αN259A, respectively. The spectral changes are marked by a single set of isosbestic points at 340, 375, 420, and 469 nm and the appearance of a sharp absorption maximum at 415 nm as well as much less pronounced maxima at 650 and 710 nm (Fig. 4). The reverse reaction, the formation of FAD from 8f-FAD at neutral or acidic pH, was not observed. The formation of 8f-FAD was independent from the buffer used (HEPES, Tris, and phosphate buffer). Similarly, removal of the hexa-histidine tag using tobacco etch virus protease had no effect on the oxidation of the 8α-methyl group.

**Figure 5.**
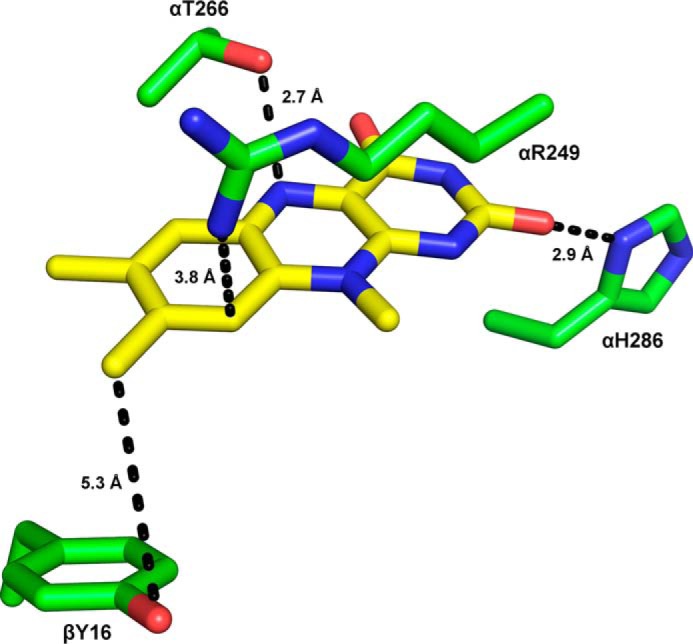
**Amino acid residues near the isoalloxazine ring system that have been targeted by site-directed mutagenesis.** αThr-266 was replaced by a methionine, αArg-249 by a cysteine, and αHis-286 by an alanine and βTyr-16 by a phenylalanine by an alanine to study their influence on 8f-FAD formation. Possible interactions of these residues with the isoalloxazine ring are indicated by the *dashed lines*.

Because the formation of 8f-FAD does not occur free in solution, we assumed that the oxidation is promoted by amino acid residues in the isoalloxazine-binding pocket. Therefore, we generated four variants of hETF featuring single amino acid replacements: αR249C, αT266M, αH286A, and βY16F (Fig. 5). Interestingly, the formation of 8f-FAD was substantially affected in all variants and decreased in the order βY16F > αT266M > αR249C (Table 1). For the αH286A variant, 8f-FAD formation could not be monitored reliably since the FAD binding was apparently compromised by the amino acid replacement. Hence, we conclude that amino acid residues directly participate in the oxidation of the 8α-methyl to the formyl group. In addition, we investigated two variants, αN259A and βE165A, that presumably increase the flexibility of the FAD-binding domain to favor the formation of a productive open conformation for electron transfer (9). Both of the variants showed a much more rapid formation of 8f-FAD, which already occurred at neutral pH (Fig. 4*B*, data for αN259A as an example). This observation strongly suggests that the conformational dynamics of hETF affect the rate of conversion of FAD to the 8f-FAD.

**Table 1:** **Formation of 8f-FAD in wildtype hETF and the variants αR249C, αT266M, and βY16F** Proteins were purified at pH 7.0 and diluted to a final concentration of 40 μm using HEPES buffer pH 8.5. Aliquots were taken and analyzed with HPLC after 0, 2, 4, 6, 8, and 24 h of incubation at 25 °C. Because the FAD cofactor was bound weakly to the αH286A variant, time-dependent formylation could not be measured reliably.

Protein	% 8f-FAD					
	0 h	2 h	4 h	6 h	8 h	24 h
WT	<2.5	15	17	21	23	47
αR249C	<2.5	<2.5	<2.5	<2.5	<2.5	<2.5
αT266M	<2.5	<2.5	<2.5	<2.5	<2.5	6
βY16F	<2.5	<2.5	<2.5	5	6	22

### Generation of 8f-FAD leads to the flavin semiquinone

Although our analysis has clearly shown the oxidation of the 8α-methyl to the formyl group, the spectral characteristics observed for the generated 8f-FAD are vastly different from those seen after release of the flavin from hETF (Fig. 2*B*). As a matter of fact, the sharp peak at 415 nm as well as the long wavelength absorption is reminiscent of a flavin semiquinone species suggesting that the 8f-FAD is present as a semiquinone radical instead of the oxidized form. The presence of a flavin radical was confirmed by EPR spectroscopy of the hETF variant αN259A, which rapidly forms the 8f-FAD at pH 8.5. A *g*-factor of 2.0048 was calculated after calibration with DPPH (*g*-factor of 2.0036) and a peak-to-peak line width of 11–13 G was measured. This line width is rather narrow for a flavin radical and suggests the stabilization of the red anionic semiquinone (10). Expectedly, hETF variant βY16F and wildtype hETF purified at pH 7.0 (no 8f-FAD formation) did not yield an EPR signal.

### Redox behavior of wildtype hETF and the αN259A variant

**Figure 6.**
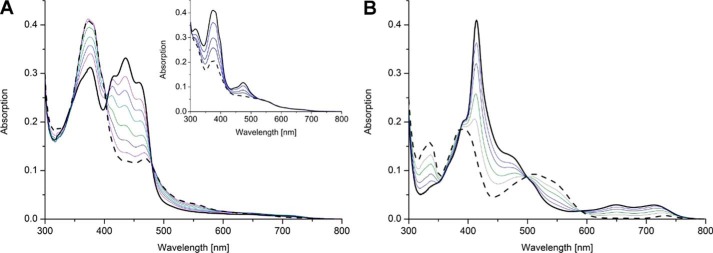
**Photoreduction of wildtype hETF and variant αN259A.** *A,* photoreduction of wildtype hETF purified at pH 7.0 proceeded to the anionic (*red*) FAD semiquinone. The semiquinone resisted further photoreduction and was completely reduced to the hydroquinone upon addition of sodium dithionite (*inset*). *B,* in contrast to wildtype hETF, photoreduction of the hETF-αN259A variant purified at pH 8.5 occurred from the radical to the hydroquinone form.

The stabilization of the anionic (red) FAD semiquinone in hETF is a well-established phenomenon (5, 6). As shown in Fig. 6*A*, photoreduction of wildtype hETF purified at neutral pH yielded the FAD semiquinone before full reduction to the hydroquinone was achieved with sodium dithionite (Fig. 6*A*, *inset*). In contrast to wildtype hETF, the αN259A variant purified at pH 8.5 was already present in the semiquinone form, and photoreduction yielded the fully reduced hydroquinone species featuring characteristic absorption maxima at 390 and 510 nm (11, 12). The hydroquinone form of 8f-FAD is sensitive to oxygen and is reoxidized to the semiquinone but not to the oxidized form. Thus, hETF not only stabilizes the semiquinone form of 8f-FAD but also prevents its oxidation by molecular oxygen. This differential behavior of FAD *versus* 8f-FAD has important implications for the one-electron transfer processes between hETF, the serviced dehydrogenases, as well as the electron acceptor hETF-QO.

### Physiological relevance of 8f-FAD, effects on stability, and protein–protein interaction

**Figure 7.**
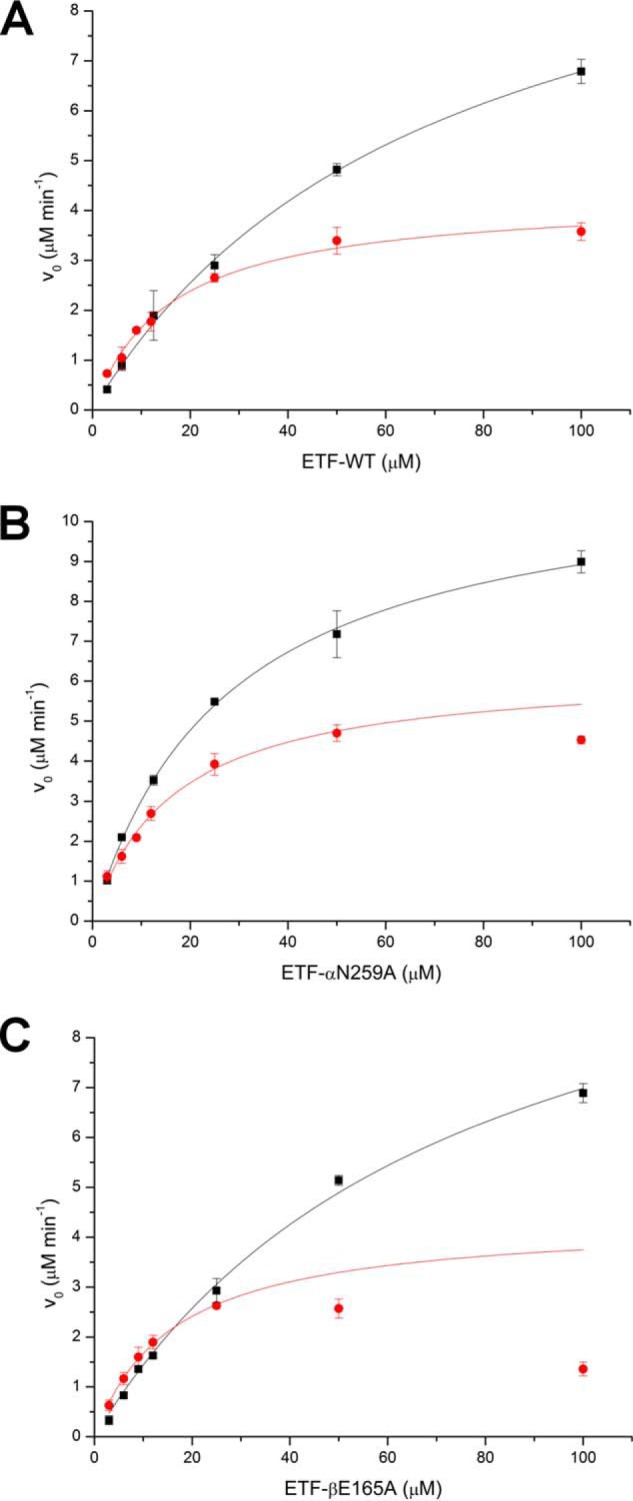
**Steady-state kinetics of wildtype hETF and the variants αN259A and βE165A purified at pH 7 (*black squares*) and pH 8.5 (*red dots*).** The measurements were performed at pH 7 with varying hETF concentrations, using hDMGDH as client dehydrogenase and DCPIP as the final electron acceptor.

To evaluate the potential impact of FAD cofactor formylation on the electron transfer reaction, we investigated the steady-state kinetics of hETF with hDMGDH, which is one of its client dehydrogenases. For that purpose, wildtype and variant αN259A were both purified in 50 mm HEPES at pH 7.0 and pH 8.5, and afterward, the steady-state kinetic parameters were determined at pH 7.0. As shown in Fig. 7 and summarized in Table 2, we observed moderate changes in the velocity of electron transfer (*k*_cat_ was reduced by ∼50%) and a 5-fold decrease in the *K_m_* values in the proteins harboring 8f-FAD. It should be noted in this context that hETF-αN259A purified at pH 7.0 already contains ∼15–30% of 8f-FAD, which may contribute to the lower *K_m_* value compared with the wildtype hETF (Table 2). In contrast, wildtype hETF purified at pH 8.5 contains only ∼30–40% 8f-FAD, and thus the obtained *K_m_* value reflects a mixed population of wildtype hETF, *i.e.* with the FAD cofactor in its methylated (native) and formylated structure, respectively. This may also contribute to the differences found between wildtype hETF and the hETF-αN259A variant (Table 2). The steady-state kinetics of protein variant βE165A purified at pH 7.0 resembled the values obtained for wildtype hETF purified at pH 7.0. In contrast to the corresponding variant αN259A, FAD formylation was absent in the freshly purified variant βE165A and was only observed during long-time storage at pH 7.

**Table 2:** **Steady-state kinetic parameters of hETF-WT and variants αN259A and βE165A** To obtain protein with a high FAD content, purification was carried out at pH 7, whereas pH 8.5 was used to maximize the 8f-FAD content. All kinetic parameters were determined at pH 7.

Protein	*K_m_*	*k*_cat_	*k*_1_
	μ*m*	*min*^−*1*^	μ*m*^−*1*^ *min*^−*1*^
WT-FAD	71 ± 5	116 ± 5	1.6 ± 0.2
WT-8f-FAD	15 ± 2	44 ± 1	2.9 ± 0.5
αN259A-FAD	30 ± 2	114 ± 2	3.8 ± 0.3
αN259A-8f-FAD	16 ± 2	63 ± 3	3.9 ± 0.8
βE165A-FAD	74 ± 9	122 ± 8	1.6 ± 0.4
βE165A-8f-FAD	16 ± 1	43 ± 2	2.7 ± 0.3

## Discussion

### Discovery of an unusual FAD derivative in an old protein

In this investigation, we have unambiguously demonstrated the formation of 8f-FAD in hETF by means of various spectroscopies such as HPLC/MS, UV-visible absorption, and NMR and EPR spectroscopy. Furthermore, it was shown that the formation of 8f-FAD strongly depended on the pH such that wildtype hETF is resistant to the formation of 8f-FAD at acidic and neutral pH, whereas at alkaline pH the slow oxidation of the 8α-methyl to the formyl group is observable (*t*_½_ = 20 h, Figs. 2*A* and 4*A*). Interestingly, 8f-FAD was not present in the oxidized but in the semiquinone state, which was stable toward oxidation by dioxygen. The UV-visible absorption spectrum featured a sharp peak at 415 nm and two minor absorption maxima at longer wavelengths (650 and 720 nm, Fig. 2*A*). The latter spectral features are clearly indicative of the (blue) neutral semiquinone, although the peak-to-peak linewidth measured by EPR spectroscopy suggested that the major fraction is the (red) anionic semiquinone. Thus, we conclude that a mixture of the blue and red semiquinone is present in hETF suggesting that our measurements were conducted near the pertinent p*K_a_* of the flavin radical, as was also found earlier for a lysine to arginine replacement in the FMN-binding pocket of lactate oxidase (13). Overall, the spectral characteristics are very similar to those reported earlier by Yorita *et al.* (13) and by Maeda *et al.* (14) for formate oxidase from *Aspergillus oryzae*. In the latter case, it was shown that oxidation of the 8α-methyl group significantly enhances enzyme activity, and thus it was argued that the 8f-FAD derivative is the cognate cofactor of the enzyme.

Because detailed studies on ETF date back to the 1950s, we were very surprised that our findings were apparently not observed previously. However, closer inspection of the literature revealed that isolation of ETF from pig liver yielded two forms (designated ETF_R_ and ETF_B_), which were reduced to the red (ETF_R_) and blue semiquinone (ETF_B_), respectively (11). Analysis of the isolated cofactors demonstrated the presence of a flavin species with absorption maxima at 463 and 352 nm, which are in fact very similar to the spectral features observed for the 8f-FAD spectrum after denaturation of wildtype hETF purified at pH 8.5 (Fig. 2*B*, *red solid line*). Furthermore, Lehman and Thorpe (11) reported the occurrence of a pink species upon reduction featuring an absorption maximum at 520 nm. Again, this is reminiscent of the absorption spectrum obtained when the αN259A hETF variant is photoreduced (Fig. 6*B*, *dashed black line*). Similarly, Yorita *et al.* (13) reported the same species upon reduction of 8-formyl FMN bound to lactate oxidase. Therefore, we assume that Lehman and Thorpe (11) have also isolated the 8f-FAD cofactor from pig liver ETF; however, neither the chemical nature of the flavin nor the mode of its generation was further investigated.

**Figure 8.**
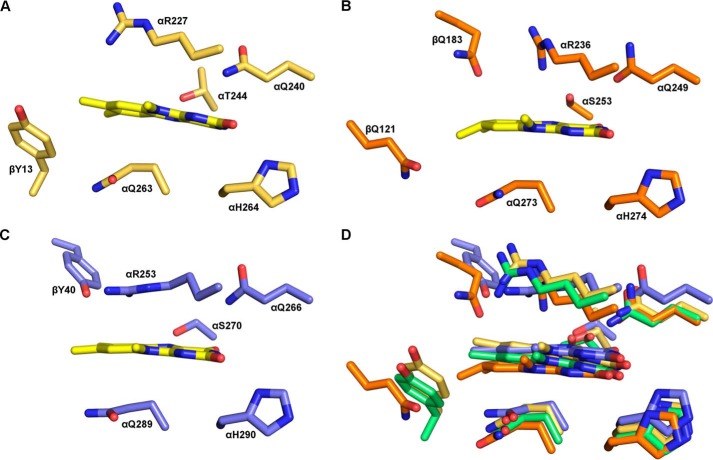
**Composition of FAD-binding site of additional ETF structures.** The crystal structures of bacterial ETFs from *P. denitrificans* (*A, gold*; PDB code 1EFP), *M. methylotrophus* (*B, orange*; PDB code 1O96), and *A. fermentans* (*C, blue*; PDB code 4KPU) were aligned with the structure of hETF (*green, D*; PDB code 1EFV). The alignment shows that all four structures have a very conserved active site composition.

**Figure 9.**
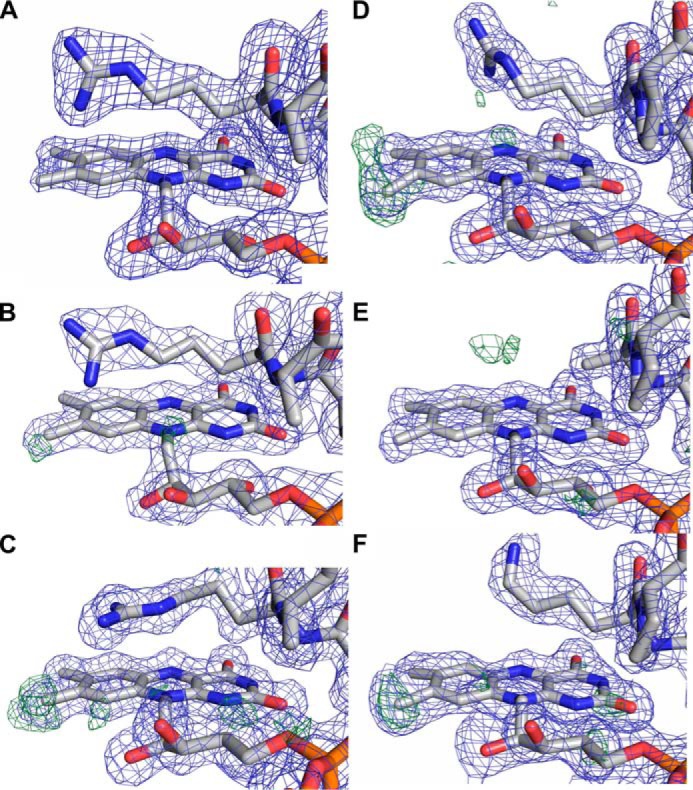
**Electron densities of the FAD region of selected ETF proteins.** *A,* human ETF (PDB code 1EFV); *B,* human ETF βE165A variant (PDB code 2A1U); *C, A. fermentans* ETF (PDB code 4KPU); *D, M. methylotrophus* ETF (PDB code 1O96); *E, M. methylotrophus* ETF αR236A variant (PDB code 3CLR); and *F, M. methylotrophus* ETF αR236K variant (PDB code 3CLU). In all panels, the 2*F_o_* − *F_c_* electron density map contoured at 1σ is shown as *light blue mesh* around the cofactor, and residues of an important loop region are on the *re* side of the flavin. The *green* density map corresponds to the *F_o_* − *F_c_* map contoured at 3σ.

Because the amino acid residues in the FAD-binding pocket of mammalian and bacterial ETFs are highly conserved (Fig. 8), it was tempting to assume that the generation of 8f-FAD is a common feature in this protein family. Thus, we have analyzed the crystal structures of previously published ETF structures, hETF (PDB code 1EFV), the ETF from *Methylophilus methylotrophus* (PDB code 1O96), *Paracoccus denitrificans* (PDB code 1EFP), and *Acidaminococcus fermentans* (PDB code 4KPU). Closer inspection of the electron densities obtained for the 8α-position showed obvious deviations from the expected electron density of a methyl group for some reported crystal structures (Fig. 9). In fact, in those cases the additional electron density is indicative of a covalent modification of the 8α-methyl group and is in line with the presence of (partially) formylated FAD (Fig. 9). With the exception of hETF, this was also found in cases where crystallization was carried out at or near pH 7, *i.e.* under conditions where oxidation of the 8α-methyl to the formyl group is very slow. The structurally very similar bacterial ETFs from *A. fermentans* and *M. methylotrophus* show clear indications of FAD modification even for wildtype forms and a variant with a conserved positive charge on the *re* side of the flavin (Fig. 9, *C, D,* and *F*), whereas no indication of 8f-FAD formation is observed for the modification-incompetent αR236A variant of *M. methylotrophus* ETF (Fig. 9*E*). With regard to ETF from *M. methylotrophus*, it is also noteworthy that Byron *et al.* (15) clearly have observed the 8f-flavin radical species (*cf*. Fig. 4) but did not further elucidate the cause of the drastic change in the absorption spectrum. The occurrence of the 8f-FAD in *M. methylotrophus* ETF is potentially relevant as this could (partially) explain the unusually positive redox potential of the protein (+196 mV for the ETF_ox_/ETF redox pair) (15), which is in accordance with previous reports that the redox potential of free 8f-FAD is ∼130–160 mV more positive than that of free FAD (8, 12).

### Proposed reaction mechanism for the formation of 8f-FAD

We propose that the oxidation of the 8α-methyl group is initiated by proton abstraction, as proposed previously (16–18), leading to the formation of a negative charge at the N(1)–C(2)=O locus of the isoalloxazine ring. After addition of water, the reduced intermediate is oxidized to yield the 8α-hydroxymethylene intermediate, which is again subject to proton abstraction at the 8α-group with the resulting enol tautomerizing to the reduced 8f-FAD. Taking the results of our mutagenesis study into account, it is conceivable that the phenolate side chain of βTyr-16 may act as a base (instead of hydroxide). Such an involvement would be reminiscent of the formation of a covalent linkage established in 8α-tyrosyl-modified flavoenzymes (19–22). The side chains of αArg-249, αThr-266, and αHis-286 support the reaction by hydrogen bonding to the N(1)–C(2)=O locus (αHis-286), N(5) (αThr-266), and by π-cation interaction (αArg-249), respectively. Interestingly, variants favoring the open conformation of hETF, *i.e.* βE165A and αN259A (see under “Discussion”), also promote the oxidation of the 8α-methyl group, probably due to greater solvent exposure of the relevant part of the isoalloxazine ring in line with the proposed role of hydroxide and water in the putative reaction mechanism. Because our experiments have shown that the generated 8f-FAD is preferentially present as the semiquinone, we propose that the 8f-FAD is rapidly reduced, *e.g. in vitro* by light and *in vivo* by one of the client dehydrogenases, to the very stable radical species.

### Effect of cofactor oxidation to the 8f-FAD on electron transfer

The oxidation of FAD to 8f-FAD clearly affected the interaction with hDMGDH, which is one of the confirmed client dehydrogenases of hETF. The most striking effect was observed on the apparent *K_m_* value defined here as the concentration of hETF at which the rate of electron transfer proceeds at half-maximal velocity. A 5-fold lower *K_m_* value was observed not only in the case of wildtype hETF but also for the βE165A and αN259A variants when 8f-FAD was present as the main cofactor moiety (Table 2). A smaller effect was also seen on the maximal rate of electron transfer from reduced hDMGDH to hETF as well as the βE165A and αN259A variants, which was ∼50–60% lower in the proteins containing 8f-FAD instead of FAD (Table 2). Thus, it is apparent that oxidation of the FAD cofactor in hETF significantly affects the interaction with hDMGDH. In fact, Toogood *et al.* (9) have reported that hETF variant βE165A has a higher affinity to hMCAD and rat DMGDH compared with the wildtype protein. Because the side chains of amino acid residues βGlu-165 and αAsn-259 stabilize a non-productive, closed hETF conformation (Scheme 1), the replacement by alanine populates the open conformation, which is conducive to complex formation with an electron delivering dehydrogenase and enabled the crystallization of a protein complex comprising hETF and hMCAD (9). As a consequence of this perturbation of the conformational equilibrium of the closed and open conformation, the protein complex of hETF with its client enzymes exhibits higher affinity and thus rationalizes the observed kinetic effects on *K_m_* value and the rate of electron transfer under steady-state conditions (Fig. 7 and Table 2).

Interestingly, formation of 8f-FAD appears to exhibit a similar effect on the interaction with client dehydrogenases as seen in the βE165A and αN259A variants. Therefore, we hypothesize that the presence of 8f-FAD in hETF leads to a significant shift of the conformational equilibrium toward the open (productive) conformation, which in turn increases the affinity to client enzymes. Currently, we are conducting hydrogen/deuterium exchange mass spectrometric experiments to test this hypothesis.

### Potential physiological role of 8f-FAD in health and disease

Considering the apparent impact of the formylation of the FAD cofactor, the central question that emerges here concerns the physiological relevance of the cofactor modification. As the pH of the mitochondrial matrix was found to be close to 8.0 (23, 24), formylation of wildtype hETF would clearly occur in this environment, albeit at a slow rate; thus, the overall lifetime of the protein will ultimately determine the fraction of hETFs containing 8f-FAD. Although, this lifetime is currently not known for hETF, mitochondrial matrix proteins were shown to exhibit half-lives in the range of 17 to more than 100 h (25). In the case that hETF belongs to the long-lived proteins, a significant fraction of the protein will in fact harbor the 8f-FAD cofactor. Moreover, it is conceivable that other proteins, such as the electron delivering dehydrogenases, promote the formation of 8f-FAD by stabilizing the open conformation of hETF as seen in the αN259A and βE165A variants.

In this context, it is very intriguing that several inborn mutations in the gene encoding human ETF are known to cause glutaric aciduria type II (GAII), also called multiple acyl-CoA dehydrogenase deficiency (OMIM entry no. 231680), a disease characterized by severe non-ketotic hypoglycemia, metabolic acidosis, and excretion of large amounts of fatty acid and amino acid-derived metabolites (26, 27). Strikingly, two of the hETF variants generated in our mutagenesis study, namely αR249C and αT266M, were reported as the cause of GAII, with the latter being the most abundant variant found in affected patients (28, 29). Because both of these variants were severely impaired in the formation of the 8f-FAD derivative in their active sites, it is tempting to speculate that the inability to catalyze the oxidation of the 8α-methyl to the formyl group contributes to the disease-causing effect of the underlying genetic mutation. In a previous study, Dwyer *et al.* (30) concluded that αArg-249 plays a crucial role in stabilizing the flavin semiquinone state; however, at that time the occurrence of 8f-FAD and its mode of generation had not been recognized.

The remarkable stability of the 8f-FAD radical may alter the operational basis of the electron transfer reactions of hETF with the serviced dehydrogenases and the terminal electron acceptor hETF-QO. In hETF with FAD as cofactor, it is assumed that the oxidized FAD receives one electron from a client dehydrogenase generating the FAD semiquinone, which is spontaneously disproportionate and subsequently reduces hETF-QO by two single electron transfer processes (29). Only the fully reduced hETF is then able to transfer two electrons to the hETF-QO. Thus, it will be interesting to see how formylation affects the electron transfer process between the hETF and the hETF-QO. In addition, 8f-FAD also possesses a much more positive redox potential, *i.e.* around −90 mV (15), and therefore formylation may also affect the rate of electron transfer between the client dehydrogenases, hETF and hETF-QO.

## Conclusions

Our study has conclusively shown that hETF catalyzes the oxidation of FAD to 8f-FAD, an unusual cofactor modification that has not been reported for hETF or any other electron-transferring flavoprotein. Furthermore, we have demonstrated that the generation of 8f-FAD strongly depends on the pH, is catalyzed by amino acid residues in the FAD-binding pocket, and is favored in variants that preferentially adopt an open conformation. Depending on the lifetime of hETF in the mitochondrial matrix, formation of 8f-FAD will be physiologically relevant in particular because the 8f-FAD alters the interaction with electron-delivering dehydrogenases and the electron acceptor hETF-QO in the inner mitochondrial membrane. It also remains to be seen whether additional factors, such as the interaction with client dehydrogenases or the hETF-QO, affect the formation of 8f-FAD. In any case, it is apparent that our discovery of the spontaneous cofactor oxidation in a central protein of mitochondrial electron handling raises important biochemical and physiological questions with implications for human health and disease.

## Experimental procedures

### Enzymes and reagents

Restriction enzymes and Phusion DNA polymerase were from Thermo Fisher Scientific (Waltham, MA), and purification columns were from GE Healthcare (Chalfont St. Giles, UK). Salt-free purified oligonucleotides for site-directed mutagenesis were synthesized by VBC-Biotech (Vienna, Austria) or Sigma. All other chemicals and media were from Carl Roth GmbH (Karlsruhe, Germany) or Sigma and were of the highest grade available.

### hETF-WT and hETF variants of gene expression design

The hETF sequence for expression of the mature hETF consisting of α- and β-subunit was designed following a similar strategy as described by Bross *et al.* (31) and was optimized for expression in *E. coli* using GeneOptimizer® (Thermo Fisher Scientific). The operon starts with a ribosomal binding site (AAGGAG), followed by a TATA box in front of the ATG start codon of the gene sequence of the β-subunit. After the stop codon of the β-subunit, a 69-bp spacer region between the β and α gene sequence was introduced, which again comprises the same ribosomal binding site and a TATA box in front of the ATG start codon of the α-subunit. The designed gene sequence was flanked by an XbaI and an XhoI restriction site and cloned for expression into a pET-28a+ vector (Thermo Fisher Scientific). In agreement with Herrick *et al.* (5), the α-subunit starts with the first amino acid of the mature αETF (αGln-20). For protein purification, a hexa-histidine tag was added to the N terminus of the β-subunit. The recombinant plasmid was transformed into *E. coli* BL21 (DE3) cells. Positive clones were selected by kanamycin resistance. Correct cloning and potent expression colonies were verified by automated sequencing. All investigated variants of hETF-WT (αR249C, αN259A, αT266M, αH286A, βY16F, and βE165A) were constructed by two-step site-directed mutagenesis with Phusion DNA polymerase and the mutation primers shown in Table 3 (the altered codons are highlighted in bold).

**Table 3:** **Primer sequences used for site-directed mutagenesis**

Primer	DNA sequence 5′–3′	*T_m_*
		°*C*
αR249C_fw	GCAGTTGGTGCAAGC**TGC**GCAGCAGTTGATGC	67.3
αR249C_rev	GCATCAACTGCTGC**GCA**GCTTGCACCAACTGC	67.3
αN259A_fw	GCAGGTTTTGTTCCG**GCT**GATATGCAGGTTGG	64.0
αN259A_rev	CCAACCTGCATATC**AGC**CGGAACAAAACCTGC	64.0
αT266M_fw	GATATGCAGGTTGGTCAG**ATG**GGCAAAATTGTTGCAC	64.8
αT266M_rev	GTGCAACAATTTTGCC**CAT**CTGACCAACCTGCATATC	64.8
αH286A_fw	CAATTCG**GCA**CTGGCAGGCATG	69.3
αH286A_rev	CCTGCCAG**TGC**CTGAATTGCACC	70.5
βY16F_fw	GTTAAACGTGTTATTGAT**TTT**GCCGTGAAAATTCGTG	60.0
βY16F_rev	CACGAATTTTCACGGC**AAA**ATCAATAACACGTTTAA	60.0
βE165A_fw	CTGAAAGTTGAACGT**GCG**ATTGATGGTGGTCTG	63.6
βE165A_rev	CAGACCACCATCAAT**CGC**ACGTTCAACTTTCAG	63.6

First, two separate PCRs using either forward or reverse primer with 10 ng of hETF-WT template DNA, 1× Phusion HF buffer, 200 μm dNTPs, 3% DMSO, 1 unit of Phusion DNA polymerase, and 0.5 μm of each primer were run in 50-μl volumes with (98 °C (2 min) − (98 °C (50 s) − 60 °C (20 s) − 68 °C (16.5 min)) × 5 − 4 °C ∞). Afterward, the separated PCRs were combined, and the same PCR program was further employed for another 20 cycles. The PCR was followed by a 2-h DpnI digestion step; the plasmid was then transformed into *E. coli* BL21 (DE3) cells, and the strain selection was done as above using the pET28a+ kanamycin resistance.

### hETF production

hETF expression was carried out in shake flasks in an HT Multitron Standard shaking system (Infors AG, Basel, Switzerland) at 150 rpm. Briefly, overnight LB medium cultures of *E. coli* BL21 (DE3) cells with the desired hETF variant were used to inoculate 1 liter of main culture in a baffled shake flask to an *A*_600_ of 0.1. After reaching an *A*_600_ of 0.6–0.8, the protein production was started by induction with 0.1 mm isopropyl 1-thio-β-d-galactopyranoside and expression took place overnight at 25 °C. The cell pellet was collected by centrifugation (2704 × *g*, 15 min, room temperature) and stored at −20 °C.

### hETF purification

In general, enzyme purification was carried out according to the following protocol. Cell lysates were prepared by four 3-min sonications (3-min cooling steps) using a Labsonic® L sonication probe (B. Braun Biotech, Berlin, Germany) in a Sonopuls® rosette cell RZ (Bandelin, Berlin, Germany). The wet cell pellet was suspended in lysis buffer (3 ml of 50 mm HEPES/NaOH, 15 mm imidazole, pH 7.0, 7.8, or 8.5 per g), and a spatula tip of FAD was added before sonication. The lysates were cleared by centrifugation (38,720 × *g*, 45 min, 4 °C) and filtration through a paper filter. Nickel ion affinity chromatography was performed by applying the cell lysates onto 5-ml HisTrap HP columns (GE Healthcare). Afterward, the columns were washed with at least 10 column volumes of lysis buffer, and the enzyme was stripped off with elution buffer (50 mm HEPES/NaOH, 200 mm imidazole, pH 7.0, 7.8, or 8.5). The purification was monitored by SDS-PAGE, and fractions containing hETF were concentrated using Amicon® ultracentrifugal filter units (10-kDa cutoff, Merck-Millipore, Darmstadt, Germany) and rebuffered to storage buffer (50 mm HEPES/NaOH, pH 7.0, 7.8, or 8.5) using Sephadex G-25 PD10 desalting columns (GE Healthcare). After rebuffering, the enzyme solution was incubated at 37 °C for 30 min and afterward cleared by centrifugation to remove aggregated protein and excessive hETF β-subunits. The obtained enzyme purity was sufficient for all kinetic and spectrophotometric studies.

### hDMGDH production and purification

hDMGDH expression and purification for use in interaction studies for steady-state kinetic analyses was performed as reported previously by Augustin *et al.* (32).

### SDS-PAGE

Enzyme samples were separated by SDS-PAGE with 12.5% separation and 5% stacking gels under reducing conditions (100 mm DTT in the sample buffer) as described by Laemmli (33). Gels were stained with Coomassie Brilliant Blue R-250 for purification control, and a PageRuler® prestained protein ladder (Thermo Fisher Scientific) was employed as protein standard.

### UV-visible absorption spectroscopy

UV-visible absorption spectra to assess protein concentration, activity, purity, and quality as well as for steady-state kinetic measurements and photoreduction were recorded with a Specord 210 spectrophotometer (Analytik Jena, Jena, Germany).

### Protein quantification and calculation of the extinction coefficient

Protein concentrations of purified hETF wildtype and variants were determined using the characteristic absorption of protein-bound FAD at 469 nm (isosbestic point of FAD and 8f-FAD). A molar extinction coefficient (ϵ) of 9900 ± 700 m^−1^ cm^−1^ for hETF was determined using the method described by Macheroux (34) based on an ϵ of free FAD at 469 nm of 9910 m^−1^ cm^−1^.

### Extraction and purification of 8f-FAD

Wildtype hETF or the variant αN259A was purified in 50 mm HEPES/NaOH buffer at pH 8.5 and concentrated to about 300–400 μm using Amico® ultracentrifugal filter units (10-kDa cutoff). After complete denaturation, which was achieved by two times treatment at 70 °C for 10 min and subsequent centrifugation at 18,500 × *g* for 10 min, the supernatants were transferred to HPLC vials for purification. HPLC purification was done on a Dionex UltiMate 3000 HPLC (Thermo Fisher Scientific) equipped with an Atlantis® dC18 column (5 μm, 4.6 × 250 mm, Waters, Milford, MA) equilibrated with H_2_O, 0.1% TFA, 7% acetonitrile, and a diode array detector for UV-visible monitoring (λ = 280, 370, 450, and 460 nm). An injection volume of 40 μl, a temperature of 25 °C, and a flow rate of 1 ml min^−1^ were used. 8f-FAD was separated from residual free FAD and other impurities for subsequent UV-visible absorption and NMR spectroscopy as well as HPLC/MS analyses using the following program: 0–25 min, 7–12% acetonitrile; 25–30 min, 95% acetonitrile; 30–35 min, 7% acetonitrile. 200-μl fractions were collected between 12.0 and 18.0 min, and fractions with a pure and typical 8f-FAD UV-visible absorption spectrum were combined. The combined fractions were dried using an ISS110 Savant SpeedVac System at 45 °C, <10 mbar vacuum, and an RH64-11 rotor (Thermo Fisher Scientific), and afterward stored at −20 °C until further use. Purity of the cofactor was controlled by HPLC measurements using the same conditions as above.

### Analysis of 8f-FAD by HPLC/ESI-MS

For HPLC/MS measurements, an Agilent Technologies 1200 series (Santa Clara, CA) equipped with a G1379B degasser, G1312B binary pump SL, G1367C HiP-ALS SL autosampler, a G1314C VWD SL UV detector, G1316B TCC SL column oven, and a G1956B MSD mass selective detector was used. The mass spectrometer was operated in positive electrospray ionization mode. The analytes were separated on an Atlantis® dC18 column (5 μm, 4.6 × 250 mm, Waters) at 25 °C by using aqueous eluent (0.1% formic acid) and acetonitrile at a flow rate of 1.0 ml min^−1^. The column was equilibrated with 7% acetonitrile in water (0.1% formic acid), and the following gradient was used for analysis: 0–2 min, 7% acetonitrile; 2–10 min, 7–100% acetonitrile; 10–12 min, 100% acetonitrile; 12–14 min, 7% acetonitrile. 10 μl of 300 μm HPLC purified 8f-FAD or 300 μm FAD solution for control, dissolved in water, were injected for each run.

### ^1^H NMR spectroscopy

4 mm solutions of purified 8f-FAD or FAD dissolved in 20% D_2_O in water (v/v) were subjected to ^1^H NMR analysis using a Varian INOVA 500 (499.82 MHz, Agilent) spectrometer. ^1^H NMR spectra were recorded at 500 MHz at 30 °C. The signal of water protons (δ_H_ 4.75 ppm) was used as the reference for the observed chemical shifts.

### EPR spectroscopy

Electron paramagnetic resonance experiments were performed with 150 μm hETF-αN259A and hETF-βY16F purified in 50 mm HEPES/NaOH, pH 8.5, and with 150 μm hETF-WT purified in 50 mm HEPES/NaOH, pH 7.0, with n X-band ECS 106 spectrometer (Bruker, Billerica, MA) with 9.45 GHz microwave frequency. A microwave power of 2 milliwatts was used, with a modulation amplitude of 2.0 G at a modulation frequency of 50 kHz. Samples were run at 295 K in 100-μl capillaries and 10 scans with a conversion time of 5.12 ms, a time constant of 10.24 ms, and a sweep time of 20.97 s. The magnetic field was scanned for 100 G from 3340 to 3440 G. The magnetic phase and field modulation amplitude of the signal channel of the EPR machine was calibrated with solid DPPH according to the manual to a *g* factor of 2.0036.

### HPLC analysis of 8f-FAD formation

Protein purified at pH 7.0 was diluted to a final concentration of 40 μm to adjust the pH to 8.5 and then incubated at 25 °C. Samples were taken after 0, (1), 2, 4, 6, 8, and 24 h, and the cofactor was isolated according to the protocol described above. After HPLC analysis, areas of the peaks corresponding to FAD and 8f-FAD were determined, and the ratio between the two chromophores was calculated.

### Steady-state kinetics

Steady-state kinetic parameters were determined spectrophotometrically according to Okamura-Ikeda *et al.* (35) using DCPIP (2,6-dichlorophenolindophenol) as terminal electron acceptor. For the assays, 125 μm DCPIP, 100 nm hDMGDH, and 0–100 μm hETF in 50 mm HEPES/NaOH, pH 7.0, were incubated at 25 °C for 10 min before the reaction was started by addition of 25 mm dimethylglycine, and the change of absorption was monitored at 600 nm for 3 min. For each concentration, at least a triplicate measurement was performed. From these data the initial velocities were determined, and *K_m_* and *k*_cat_ values were assessed using a non-linear hyperbolic fit in Origin 8.6 (OriginLab Corp., Northampton, MA).

### Anaerobic photoreduction

Photoreduction of flavoproteins was done according to the method reported by Massey *et al.* (36). The experimental procedure of photoreduction and reoxidation was performed as described by Augustin *et al.* (32). Approximately 20 μm purified enzyme in 50 mm HEPES/NaOH, pH 7.0, was reduced at 15 °C. Further reduction of hETF-WT was achieved by adding a 10-fold excess of sodium dithionite to the solution.

### Thermo FAD thermal stability

The temperature stability of the proteins was determined by monitoring the change in the intrinsic protein fluorescence of FAD in a Thermofluor® assay (37). Thermo FAD measurements were carried out with an FX Connect real-time PCR system (Bio-Rad) in 25 μl of 50 mm HEPES/NaOH, pH 7.0, and 30 μm enzyme. The samples were pre-heated to 25 °C, and then the temperature was increased in 0.5 °C/min steps to 95 °C. Fluorescence data were collected using the FRET channel. Melting temperatures (*T_m_*) were determined using the CFX Manager 3.0 software (Bio-Rad).

## Author contributions

P. A., M. T., A. W., and P. M. conceptualization; P. A., M. T., K. F., and R. P. data curation; P. A., M. T., K. F., E. C. G., R. P., A. W., and P. M. formal analysis; P. A., M. T., K. F., E. C. G., and P. M. validation; P. A., M. T., R. P., and A. W. visualization; P. A. and P. M. writing-original draft; P. A., M. T., A. W., and P. M. writing-review and editing; A. W. and P. M. methodology; P. M. resources; P. M. supervision; P. M. funding acquisition; P. M. investigation; P. M. project administration.

## Footnotes

3 The abbreviations used are:

ETF: electron-transferring flavoprotein
8f-FAD: 8-formyl-FAD
h: human
DMGDH: dimethylglycine dehydrogenase
DPPH: α,α′-diphenyl-β-picrylhydrazyl
ETF-QO: ETF-ubiquinone oxidoreductase
GAII: glutaric aciduria type II
MCAD: medium chain acyl-CoA dehydrogenase
PDB: Protein Data Bank
Ni-NTA: nickel-nitrilotriacetic acid
a.u.: atomic unit.

## References

[1] Crane F. L., Mii S., Hauge J. G., Green D. E., and Beinert H. (1956) On the mechanism of dehydrogenation of fatty acyl derivatives of coenzyme A. I. The general fatty acyl coenzyme A dehydrogenase. J. Biol. Chem. 218, 701–706 13295224

[2] Toogood H. S., Leys D., and Scrutton N. S. (2007) Dynamics driving function–new insights from electron-transferring flavoproteins and partner complexes. FEBS J. 274, 5481–5504 10.1111/j.1742-4658.2007.06107.x 17941859

[3] Ghisla S., and Thorpe C. (2004) Acyl-CoA dehydrogenases: a mechanistic overview. Eur. J. Biochem. 271, 494–508 10.1046/j.1432-1033.2003.03946.x 14728676

[4] Toogood H. S., van Thiel A., Basran J., Sutcliffe M. J., Scrutton N. S., and Leys D. (2004) Extensive domain motion and electron transfer in the human electron-transferring flavoprotein·medium chain acyl-CoA dehydrogenase complex. J. Biol. Chem. 279, 32904–32912 10.1074/jbc.M404884200 15159392

[5] Herrick K. R., Salazar D., Goodman S. I., Finocchiaro G., Bedzyk L. A., and Frerman F. E. (1994) Expression and characterization of human and chimeric human-*Paracoccus denitrificans* electron transfer flavoproteins. J. Biol. Chem. 269, 32239–32245 7798224

[6] Husain M., and Steenkamp D. J. (1983) Electron transfer flavoprotein from pig liver mitochondria. A simple purification and re-evaluation of some of the molecular properties. Biochem. J. 209, 541–545 10.1042/bj2090541 6847633 PMC1154123

[7] Horai H., Arita M., Kanaya S., Nihei Y., Ikeda T., Suwa K., Ojima Y., Tanaka K., Tanaka S., Aoshima K., Oda Y., Kakazu Y., Kusano M., Tohge T., Matsuda F., et al (2010) MassBank: a public repository for sharing mass spectral data for life sciences. J. Mass Spectrom. 45, 703–714 10.1002/jms.1777 20623627

[8] Doubayashi D., Ootake T., Maeda Y., Oki M., Tokunaga Y., Sakurai A., Nagaosa Y., Mikami B., and Uchida H. (2011) Formate oxidase, an enzyme of the glucose-methanol-choline oxidoreductase family, has a His-Arg pair and 8-formyl-FAD at the catalytic site. Biosci. Biotechnol. Biochem. 75, 1662–1667 10.1271/bbb.110153 21897046

[9] Toogood H. S., van Thiel A., Scrutton N. S., and Leys D. (2005) Stabilization of non-productive conformations underpins rapid electron transfer to electron-transferring flavoprotein. J. Biol. Chem. 280, 30361–30366 10.1074/jbc.M505562200 15975918

[10] Schleicher E., Bittl R., and Weber S. (2009) New roles of flavoproteins in molecular cell biology: blue-light active flavoproteins studied by electron paramagnetic resonance. FEBS J. 276, 4290–4303 10.1111/j.1742-4658.2009.07141.x 19624734

[11] Lehman T. C., and Thorpe C. (1992) A new form of mammalian electron-transferring flavoprotein. Arch. Biochem. Biophys. 292, 594–599 10.1016/0003-9861(92)90036-V 1731621

[12] Edmondson D. E. (1974) Intramolecular hemiacetal formation in 8-formylriboflavine. Biochemistry 13, 2817–2821 10.1021/bi00711a006 4407611

[13] Yorita K., Matsuoka T., Misaki H., and Massey V. (2000) Interaction of two arginine residues in lactate oxidase with the enzyme flavin: conversion of FMN to 8-formyl-FMN. Proc. Natl. Acad. Sci. U.S.A. 97, 13039–13044 10.1073/pnas.250472297 11078532 PMC27174

[14] Maeda Y., Doubayashi D., Oki M., Nose H., Sakurai A., Isa K., Fujii Y., and Uchida H. (2009) Expression in *Escherichia coli* of an unnamed protein gene from *Aspergillus oryzae* RIB40 and cofactor analyses of the gene product as formate oxidase. Biosci. Biotechnol. Biochem. 73, 2645–2649 10.1271/bbb.90497 19966484

[15] Byron C. M., Stankovich M. T., Husain M., and Davidson V. L. (1989) Unusual redox properties of electron-transfer flavoprotein from *Methylophilus methylotrophus*. Biochemistry 28, 8582–8587 10.1021/bi00447a047 2605209

[16] Jhulki I., Chanani P. K., Abdelwahed S. H., and Begley T. P. (2016) A remarkable oxidative cascade that replaces the riboflavin C8 methyl with an amino group during roseoflavin biosynthesis. J. Am. Chem. Soc. 138, 8324–8327 10.1021/jacs.6b02469 27331868 PMC5610575

[17] Robbins J. M., Souffrant M. G., Hamelberg D., Gadda G., and Bommarius A. S. (2017) Enzyme-mediated conversion of flavin adenine dinucleotide (FAD) to 8-formyl FAD in formate oxidase results in a modified cofactor with enhanced catalytic properties. Biochemistry 56, 3800–3807 10.1021/acs.biochem.7b00335 28640638

[18] Konjik V., Brünle S., Demmer U., Vanselow A., Sandhoff R., Ermler U., and Mack M. (2017) The crystal structure of RosB: Insights into the reaction mechanism of the first member of a family of flavodoxin-like enzymes. Angew. Chem. Int. Ed. Engl. 56, 1146–1151 10.1002/anie.201610292 27981706

[19] Mewies M., McIntire W. S., and Scrutton N. S. (1998) Covalent attachment of flavin adenine dinucleotide (FAD) and flavin mononucleotide (FMN) to enzymes: The current state of affairs. Protein Sci. 7, 7–20 10.1002/pro.5560070102 9514256 PMC2143808

[20] McIntire W., Edmondson D. E., Singer T. P., and Hopper D. J. (1981) 8α-(o-Tyrosyl)flavin adenine dinucleotide, the prosthetic group of bacterial *p*-cresol methylhydroxylase. Biochemistry 20, 3068–3075 10.1021/bi00514a013 7248267

[21] Kim J., Fuller J. H., Cecchini G., and McIntire W. S. (1994) Cloning, sequencing, and expression of the structural genes for the cytochrome and flavoprotein subunits of *p*-cresol methylhydroxylase from two strains of *Pseudomonas putida*. J. Bacteriol. 176, 6349–6361 10.1128/jb.176.20.6349-6361.1994 7929007 PMC196977

[22] Reeve C. D., Carver M. A., and Hopper D. J. (1989) The purification and characterization of 4-ethylphenol methylenehydroxylase, a flavocytochrome from *Pseudomonas putida* JD1. Biochem. J. 263, 431–437 10.1042/bj2630431 2556994 PMC1133447

[23] Porcelli A. M., Ghelli A., Zanna C., Pinton P., Rizzuto R., and Rugolo M. (2005) pH difference across the outer mitochondrial membrane measured with a green fluorescent protein mutant. Biochem. Biophys. Res. Commun. 326, 799–804 10.1016/j.bbrc.2004.11.105 15607740

[24] Llopis J., McCaffery J. M., Miyawaki A., Farquhar M. G., and Tsien R. Y. (1998) Measurement of cytosolic, mitochondrial, and Golgi pH in single living cells with green fluorescent proteins. Proc. Natl. Acad. Sci. U.S.A. 95, 6803–6808 10.1073/pnas.95.12.6803 9618493 PMC22642

[25] Hare J. F., and Hodges R. (1982) Turnover of mitochondrial matrix polypeptides in hepatoma monolayer cultures. J. Biol. Chem. 257, 12950–12953 7130189

[26] Christensen E., Kølvraa S., and Gregersen N. (1984) Glutaric aciduria type II: Evidence for a defect related to the electron transfer flavoprotein or its dehydrogenase. Pediatr. Res. 18, 663–667 10.1203/00006450-198407000-00020 6433313

[27] Frerman F. E., and Goodman S. I. (2001) in The Metabolic and Molecular Bases of Inherited Disease (Scriver C. R., Beaudet A. L., Sly W. S., and Valle D., eds) 8th Ed., pp. 2357–2365, McGraw-Hill, New York

[28] Schiff M., Froissart R., Olsen R. K., Acquaviva C., and Vianey-Saban C. (2006) Electron transfer flavoprotein deficiency: functional and molecular aspects. Mol. Genet. Metab. 88, 153–158 10.1016/j.ymgme.2006.01.009 16510302

[29] Salazar D., Zhang L., deGala G. D., and Frerman F. E. (1997) Expression and characterization of two pathogenic mutations in human electron transfer flavoprotein. J. Biol. Chem. 272, 26425–26433 10.1074/jbc.272.42.26425 9334218

[30] Dwyer T. M., Zhang L., Muller M., Marrugo F., and Frerman F. (1999) The functions of the flavin contact residues, αArg249 and βTyr16, in human electron transfer flavoprotein. Biochim. Biophys. Acta 1433, 139–152 10.1016/S0167-4838(99)00139-9 10446367

[31] Bross P., Pedersen P., Winter V., Nyholm M., Johansen B. N., Olsen R. K., Corydon M. J., Andresen B. S., Eiberg H., Kolvraa S., and Gregersen N. (1999) A polymorphic variant in the human electron transfer flavoprotein α-chain (α-T171) displays decreased thermal stability and is overrepresented in very-long-chain acyl-CoA dehydrogenase-deficient patients with mild childhood presentation. Mol. Genet. Metab. 67, 138–147 10.1006/mgme.1999.2856 10356313

[32] Augustin P., Hromic A., Pavkov-Keller T., Gruber K., and Macheroux P. (2016) Structure and biochemical properties of recombinant human dimethylglycine dehydrogenase and comparison to the disease-related H109R variant. FEBS J. 283, 3587–3603 10.1111/febs.13828 27486859 PMC5082570

[33] Laemmli U. K. (1970) Cleavage of structural proteins during the assembly of the head of bacteriophage T4. Nature 227, 680–685 10.1038/227680a0 5432063

[34] Macheroux P. (1999) UV-visible spectroscopy as a tool to study flavoproteins. Methods Mol. Biol. 131, 1–7 10494538 10.1385/1-59259-266-X:1

[35] Okamura-Ikeda K., Ikeda Y., and Tanaka K. (1985) An essential cysteine residue located in the vicinity of the FAD-binding site in short-chain, medium-chain, and long-chain acyl-CoA dehydrogenases from rat liver mitochondria. J. Biol. Chem. 260, 1338–1345 3968065

[36] Massey V., Hemmerich P., Knappe W. R., Duchstein H. J., and Fenner H. (1978) Photoreduction of flavoproteins and other biological compounds catalyzed by deazaflavins. Appendix: photochemical formation of deazaflavin dimers. Biochemistry 17, 9–16 618539

[37] Forneris F., Orru R., Bonivento D., Chiarelli L. R., and Mattevi A. (2009) Thermo FAD, a Thermofluor®-adapted flavin *ad hoc* detection system for protein folding and ligand binding. FEBS J. 276, 2833–2840 10.1111/j.1742-4658.2009.07006.x 19459938

